# Flow-Field Characteristics of High-Temperature Annular Buoyant Jets and Their Development Laws Influenced by Ventilation System

**DOI:** 10.1155/2013/826514

**Published:** 2013-08-13

**Authors:** Yi Wang, Yanqiu Huang, Jiaping Liu, Hai Wang, Qiuhan Liu

**Affiliations:** School of Environmental and Municipal Engineering, Xi'an University of Architecture and Technology, No. 13 Yanta Road, Xi'an, Shaanxi 710055, China

## Abstract

The flow-field characteristics of high-temperature annular buoyant jets as well as the development laws influenced by ventilation system were studied using numerical methods to eliminate the pollutants effectively in this paper. The development laws of high-temperature annular buoyant jets were analyzed and compared with previous studies, including radial velocity distribution, axial velocity and temperature decay, reattachment position, cross-section diameter, volumetric flow rate, and velocity field characteristics with different pressures at the exhaust hood inlet. The results showed that when the ratio of outer diameter to inner diameter of the annulus was smaller than 5/2, the flow-field characteristics had significant difference compared to circular buoyant jets with the same outer diameter. For similar diameter ratios, reattachment in this paper occurred further downstream in contrast to previous study. Besides, the development laws of volumetric flow rate and cross-section diameter were given with different initial parameters. In addition, through analyzing air distribution characteristics under the coupling effect of high-temperature annular buoyant jets and ventilation system, it could be found that the position where maximum axial velocity occurred was changing gradually when the pressure at the exhaust hood inlet changed from 0 Pa to −5 Pa.

## 1. Introduction

Inside the industrial buildings, most of production technologies cannot completely prevent noxious substances from sending out to the air. High-temperature heat source induced annular buoyant jets in large space widely exists in the iron and steel, coking, and machinery industries. For examples, it may be produced by pouring molten iron into filling tank in blast furnace plant or dumping steel slag in deslagging plant. During the process of dumping high-temperature materials, strong shear force may be produced under impact and squeezing action. Induced by the shear airflow and high-temperature heat source, the shear force makes the dusty airflow generate a strong annular buoyant jet upward. Although the annular buoyant jet shows similar law to that of circular buoyant jet when developed at a certain stage, significant differences still exist between them in space relevant to industrial production.

Earlier studies on annular jets included the work of Maki and Yabe [1] and Maki and Ito [2]. Maki and Yabe [1] performed experiments on annular turbulent jets. They observed that reverse stagnation point might occur mainly depending upon the jet height in the flow field. While, some works on liquid annular jet were reported by Ramos [3, 4], extensive studies on annular air jets were conducted using theoretical analysis and experiments. Aly and Rashed [5] experimentally studied mean velocity and turbulence intensities of an annular jet of small width issuing into stagnant surroundings. Warda et al. [6] investigated the near-field region characteristics of free turbulent circular central and annular jets using Laser Doppler Anemometry. Furthermore, Kitmura and Sumita [7] reported the results of laboratory experiments on a turbulent plume; a simplified model to study how the shape of the plume changes as a function of time. At the same time, the flow-field characteristics of annular buoyant jets received great attention from computational fluid dynamics (CFD). Mollendorf and Gebhart [8] adopted numerical solution to analyze the effect of a small amount of thermal buoyancy on the velocity and temperature fields of a circular, laminar, and vertical jet. In addition, Chen and Nikitopoulos [9] used a differential model to investigate the near-field characteristics of buoyant jets discharging into a stagnant uniform environment. And then the *k-e* model of turbulence was used for calculating dynamical and thermal fields in plane turbulent vertical jets in a uniform stagnant environment [10]. Numerical investigations were also performed to predict heat transfer characteristics of laminar annular jets impinging on a surface [11]. As indicated by Shuja et al. [12], annular jet impingement onto a conical cavity and heat transfer rates from the cavity surfaces were examined for various jet velocities, two outer angles of the annular nozzle, and two cavity depths. Afterwards, El-Amin et al. [13] studied the problem of low-density gas jet injected into high-density ambient numerically which was important and common in practical applications. Through comparison and analyses, the following conclusions could be drawn. The flow-field characteristics of annular buoyant jets under some typical working conditions could be revealed by conducting experiment, but the experiment could not describe accurately the specific changes of flow-field under different working conditions; therefore, the application condition was limited. The practical technologies in industrial production were of great difference, and the flow-field characteristics might vary under different working conditions. Almost all of the previous researchers analyzed the characteristics of annular jets using experiment methods or numerical methods. Some focused on the isothermal annular jets designed in laboratory, and some focused on the airflow characteristics themselves. In practical situations, the ventilation system has great impacts on the flow-field characteristics of annular jets associated with great initial buoyancy fluxes. However, related studies are pretty lacking and only suitable for the particular conditions in the open literatures. In order to improve the control efficiency of exhaust hood in the treatment of industrial pollutants under different technological conditions, the flow-field characteristics of high-temperature annular buoyant jets as well as the development laws influenced by ventilation system were studied using numerical methods in this paper.

The inner diameter of the annular buoyant jets which simplified the high-temperature materials dumping process in practical industrial production was related to the area of dumped materials. Initial velocity of the buoyant jets was directly determined by the materials dumping velocity, and variety of dumped materials determined the initial temperature of the annular buoyant jets. Having a good knowledge of the flow-field characteristic under the coupling effect of high-temperature annular buoyant jets and ventilation system was beneficial to eliminate the pollutants effectively. Therefore, the influences of the width of the high-temperature annular buoyant jets, initial parameters, and the pressure at the exhaust hood inlet on flow-field characteristics of high-temperature annular buoyant jets were studied in this paper.

## 2. Materials and Methods

The computational fluid dynamics (CFD) software Fluent was used in this paper to work out the governing equations in order to study the flow characteristics of high-temperature annular buoyant jets and the air distribution characteristics under the coupling effect of high-temperature annular buoyant jets and ventilation system under different industrial working conditions.

### 2.1. Airflow Model

The airflow-field was modelled by solving the Navier-Stokes equations based on the finite volume method. Turbulence models were also needed to assess the effects of turbulence on momentum and heat transfer. Historically, there have been numerous efforts to establish turbulence models for various applications. The *k-e* type two-equation turbulence models were computationally more efficient and stable than complicated Reynolds stress models. This paper used Realizable *k-e* turbulence model combined with the standard wall function to simulate the three-dimensional airflow field. The realizable *k-e* model was similar in form to the standard *k-e* model but included several refinements, which made it more accurate and reliable for a wider class of flows than the standard *k-e* model [14]. Viscous heating and full buoyancy effect were all adopted to obtain accurate results for high-temperature annular buoyant jets. Incompressible-ideal-gas law was adopted to reflect the change of air density to temperature in the momentum equations. The convection and diffusion terms in the Navier-Stokes equations for all variables except for pressure were discretized by second-order upwind scheme. The pressure term was discretized by Pressure Staggering Option Scheme. The Semi-Implicit Method for Pressure-Linked Equations (SIMPLE) algorithm was used to couple the pressure and velocity variables. The simulations were carried out with commercially available CFD code FLUENT 6.3.

### 2.2. Computational Domain and Boundary Conditions

**Figure 1:**
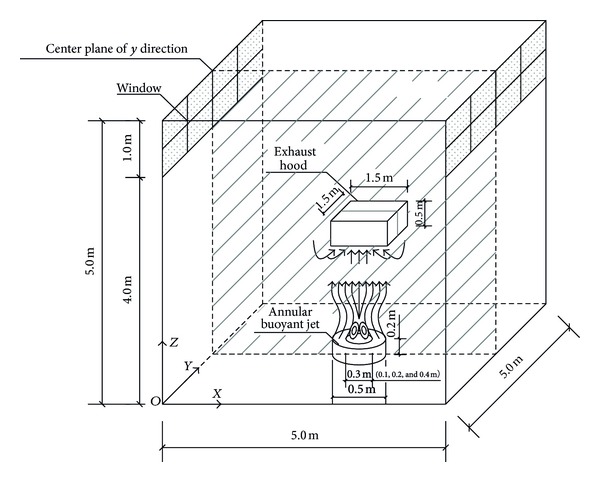
Configuration of the simulated chamber.

Based on parameters of experimental conditions from Sha [13], the verification model was established to verify the reliability of the numerical simulation model. The geometry of the simulated chamber with high-temperature annular buoyant jets was shown in Figure 1. Its dimension was 5 m (length) × 5 m (width) × 5 m (height). The origin of the coordinate system was also marked in Figure 1. The size of the left window was the same as the right, 5 m × 1 m. The inner diameter of the annular, *D*_*i*_, was 0.3 m. The outer diameter of the annular, *D*_*o*_, was 0.5 m. And its height was 0.2 m. The objects and corresponding sizes mentioned previously were referred to as the basic model in this study. To compare simulation results with the experimental data [15], the initial velocity, *U*_0_, assumed to be uniform was set as 1.2 m/s, and the initial temperature, *t*_0_, assumed to be constant was set as 95°C.

Tetrahedral mesh elements with the first grid at 20 mm away from the annular were generated in the region of the chamber. Grid size was increasing gradually at a ratio of 1.05, and the maximum was 200 mm. Based on the grid-independence test, 1042459 tetrahedral mesh elements using GAMBIT mesh tool generated in the computational domain were sufficiently fine to find out the flow-field characteristics. The boundary condition of the window was “pressure outlet.” The walls of the chamber were assumed to be hydraulic smooth and thermal isolation. Taking the experimental conditions into consideration, the turbulence intensity was set as 20%.

### 2.3. Simulation Validation

**Figure 2:**
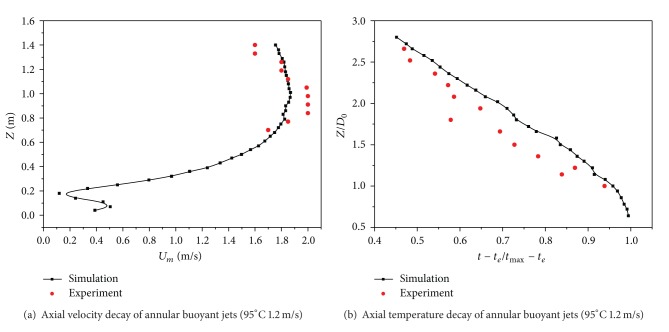
Validation of the computational simulations.

The model adopted in this paper was a simplified model for experiment, and the boundary conditions of numerical simulation were arranged as ideal conditions; therefore, there were some differences between the set conditions and the practical physical conditions. *U* was the axial velocity across the jet, m/s; *U*_max⁡_ was the maximum axial velocity, m/s. Defining a nondimensional jet temperature decay ratio (*t* − *t*_*e*_)/(*t*_max⁡_ − *t*_*e*_), where *t* was the axial temperature across the jet, °C; *t*_max⁡_ was the maximum axial temperature, °C; *t*_*e*_ was the environment temperature, namely, room temperature, °C, and by comparing the experimental data of Sha [13] with numerical simulation results of this paper, it could be found that the maximum error of axial velocities between the simulation results and the experimental data was 11.12% and the minimum error was 0.11%, whereas the maximum error of axial temperature between them was 26.60% and the minimum error was 2.11%. The validation of the numerical results for high-temperature annular buoyant jets was provided by the comparison with experimental data of Sha [13] in Figure 2.

## 3. Results and Discussion

### 3.1. Influence of Width of High-Temperature Annular Buoyant Jets

In the practical production technologies, the inner diameters of heat sources were different, which had an effect on the flow field of high-temperature annular buoyant jets. Five working conditions were selected in this paper for numerical simulation analysis. In these conditions, only the width of the high-temperature annular buoyant jets was different from each other. The initial velocity was set as 1.2 m/s, the initial temperature was set as 95°C, and both of them remained constant. The five annular heat sources with different dimensions were set as follows: *D*_0_ = 0.50 m *D*_*i*_ = 0.40 m, *D*_0_ = 0.50 m *D*_*i*_ = 0.30 m, *D*_0_ = 0.50 m *D*_*i*_ = 0.20 m, *D*_0_ = 0.50 m *D*_*i*_ = 0.10 m, and *D*_0_ = 0.50 m *D*_*i*_ = 0 m (namely, circular jet). The flow-field characteristics of high-temperature annular buoyant jets with different jet widths were analyzed and discussed as given afterwards.

#### 3.1.1. Radial Velocity Distribution of Four Annular Jet Widths

**Figure 3:**
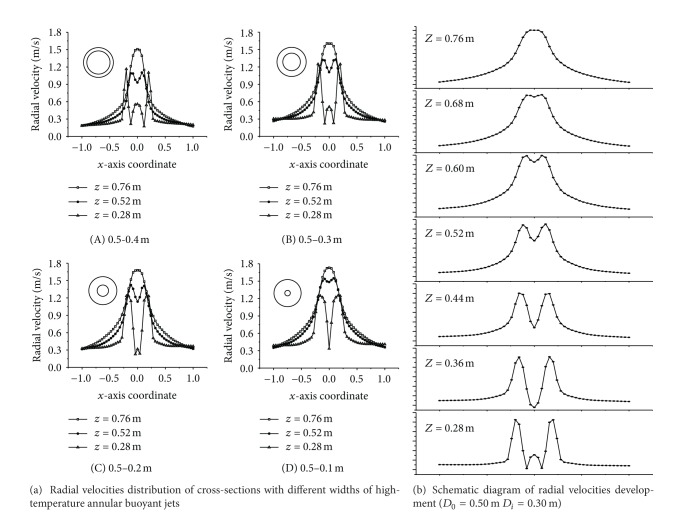
Variation of radial velocity distribution of four annular jet widths.

Two peak velocities occurred in the process of high-temperature annular buoyant jets development. The middle peak velocity (seen in Figure 3) was mainly caused by the vortex with buoyant jets at the sides expanding to the center, while peak velocities at the sides were the common characteristics of the buoyant jets; namely, the axial velocity of buoyant jets reached the maximum gradually when acceleration action due to buoyancy and velocity decay due to entrainment were in equilibrium. The velocities of three cross-sections, namely, *Z* = 0.28 m, *Z* = 0.52 m, and *Z* = 0.76 m, were selected to reflect the differences of radial velocities distribution of cross-sections with different widths of high-temperature annular buoyant jets, as shown in Figure 3(a).

It could be seen from Figure 3(a) that the middle peak velocity gradually decreased and disappeared eventually with the increase of width of high-temperature annular buoyant jets. When the outer diameter and inner diameter of the annulus were 0.5 m and 0.4 m, respectively, the vortex strength was relatively intensive and gave rise to high velocity because of the small jet width and the sudden area contraction. When the outer diameter and inner diameter of the annulus were 0.5 m and 0.1 m, respectively, the high-temperature annular buoyant jets expanded gently to the center from both sides and induced small vortex disturbance; therefore, the peak velocity due to the vortex was relatively small. Taking annulus with 0.5 m of outer diameter and 0.3 m of inner diameter, for example, the development of radial velocity was shown in Figure 3(b). It showed that, for a jet from an annular slot, the annular flow merged towards the axis of the annulus forming a velocity profile further downstream similar to that for a circular jet.

#### 3.1.2. Axial Velocity and Temperature Decay of Different Annular Jet Widths

**Figure 4:**
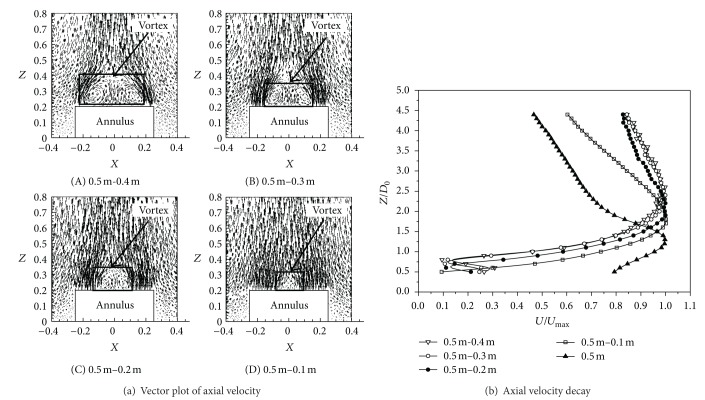
Axial velocity decay of different annular jet widths.

According to Ko and Chan [16], the flow pattern could be divided into three zones, an initial merging zone, an intermediate zone, and a fully developed zone. The initial merging zone was the nearest to the nozzle exit, and the length was very short. The intermediate zone came immediately downstream of the initial merging zone. Then a complete merging of the flows from the initial merging zone, namely, the fully merged zone, occurred. The mixing flows of the annular potential core were both from the outer mixing region and associated with the central axis of the nozzle in the intermediate zone. In the fully developed zone, the flow behaved like a combined jet with characteristics similar to those of a single circular jet. Axial velocity decay of different annular jet widths was depicted in Figure 4.

It could be clearly seen from Figure 4 that the axial velocity of high-temperature annular buoyant jets with different jet widths had the same development law; namely, it increased firstly, then decreased, and repeated. In the fully developed zone, decay rates of axial velocity were almost the same when the ratios of outer diameter to inner diameter of the annulus were 5/4, 5/3, and 5/2. While when the ratio was 5/1, the decay rate of axial velocity was close to that of circle with the same outer diameter. Hence, we could take the high-temperature annular buoyant jets with 5/1 ratio of outer diameter to inner diameter as the circular buoyant jets with the same outer diameter and employ the development laws of circular buoyant jets for those of annulus. Besides, the 5/2 ratio of outer diameter to inner diameter of annulus could be regarded as the limit of ratio of outer diameter to inner diameter for simplifying the high-temperature annular buoyant jets to the circular ones.

**Figure 5:**
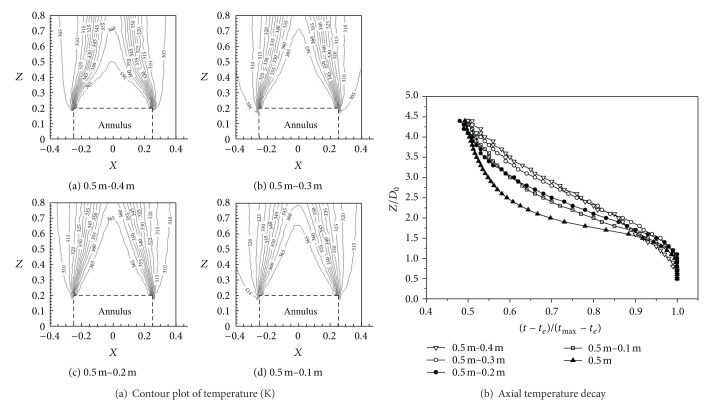
Axial temperature decay of different annular jet widths.

The temperature decay regions of annular buoyant jets were similar to the velocity decay regions; however, the extents of these regions were different from those of velocity decay, and these regions usually occurred before the corresponding velocity regions. Because energy diffusion was more extensive than momentum diffusion, it was clear that the temperature profile was flatter than the velocity profile. Axial temperature decay of different annular jet widths was shown in Figure 5.

#### 3.1.3. Reattachment Position of Four Annular Jet Widths

The axial mean velocity component along the centreline started to grow until the maximum reached at different *Z*/*D*_0_ for different annular jet widths. This location represented the reattachment point, that is, the point at which the high velocity flow which was inherited from the annular potential core met at the centreline [16]. In other words, it was the point where the location of the maximum annular velocity reached the centreline of the jet configuration.

**Figure 6:**
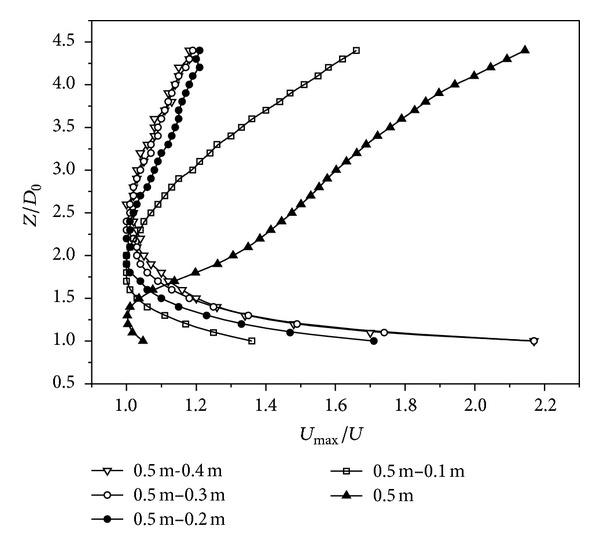
The reattachment point of different annular jet widths.

As shown in Figure 6, the hypothetical origin of the jet lied at different locations. The reattachment points for *D*_0_ = 0.50 m *D*_*i*_ = 0.40 m, *D*_0_ = 0.50 m *D*_*i*_ = 0.30 m, *D*_0_ = 0.50 m *D*_*i*_ = 0.20 m, and *D*_0_ = 0.50 m *D*_*i*_ = 0.10 m correspondingly occurred at *Z*/*D*_0_ = 2.60, 2.40, 1.90, and 1.80.

Aly and Rashed [5] provided that the reattachment occurred at 1.18*D*_0_ with 3 mm jet width. Ko and Chan [16] correlated the reattachment distance with the nozzle diameter ratio *D*_0_/*D*_*i*_. According to this correlation, the reattachment occurred at 0.8*D*_0_ with 170 mm jet width. In their paper, they mentioned that the reattachment of the jet of Miller and Comings was found to be 1.47*D*_0_ with 170 mm jet width, while the point of reattachment occurred at 1.90*D*_0_ with 150 mm jet width; and it was 1.80*D*_0_ with 200 mm jet width in this paper. Chigier and Bear [17] found that the reattachment occurred at an axial position of 2.06*D*_0_ with 97 mm jet width. However, it was 2.40*D*_0_ with 100 mm jet width in the present study. In summary, for similar diameter ratios, reattachments in this paper occurred further downstream in contrast to previous study. This phenomenon might be due to the strong buoyancy force effects on the hot air jets.

### 3.2. Influence of Initial Parameters of High-Temperature Annular Buoyant Jets

In the practical industrial production, the initial velocity of high-temperature annular buoyant jets was closely related to the dumping velocity, and the initial temperature was dependent on the variety of dumped materials. The diffusion of the high-temperature annular buoyant jets would be influenced by buoyancy forces as well as inertia forces due to jet momentum. Different initial parameters reflected the relative strength of buoyancy forces and inertia forces. And they were shown in Table 1. The flow-field, volumetric flow rate, and diameter of cross-section varying along with the height influenced by the initial parameters would be discussed in the following sections in turn.

#### 3.2.1. Axial Velocity and Temperature Decay with Different Initial Parameters

**Figure 7:**
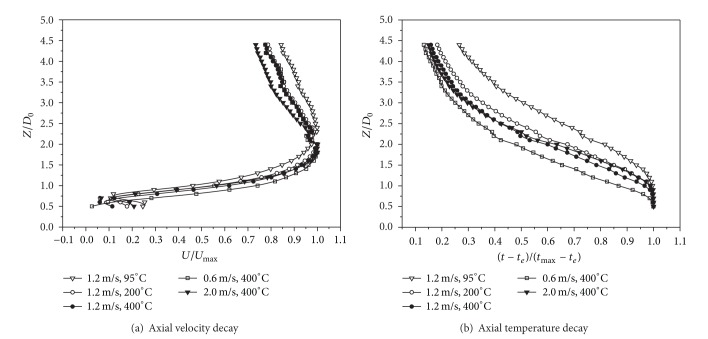
Comparison of axial velocity and temperature decay with different initial parameters of annular buoyant jets.

The decay laws of axial velocity and axial temperature with the same initial velocity and different initial temperature as well as with the different initial velocity and same initial temperature were shown in Figure 7. From Figure 7(a), it was found that, after the reattachment point, axial velocity decay was growing intensively with the initial temperature increasing at a constant initial velocity and with the initial velocity increasing at a constant initial temperature except for Case 3. From Figure 7(b), we could see that, after the reattachment point, axial temperature decay was growing intensively with the initial temperature increasing at a constant initial velocity and with the initial velocity decreasing at a constant initial temperature except for Case 3. The special development laws of Case 3 would be discussed in the next section in details.

#### 3.2.2. Development Laws of Volumetric Flow Rate with Different Initial Parameters

**Figure 8:**
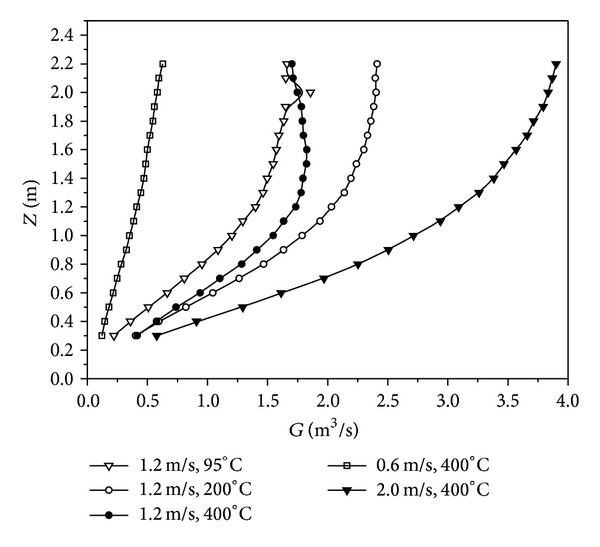
Volumetric flow rate of high-temperature annular buoyant jets at cross-section varying along with the height with different initial parameters.

The development law of volumetric flow rate (*G*) of high-temperature annular buoyant jets at cross-section varying along with the height could provide some references for the exhaust air rate, size, and installation height of exhaust hood. The volumetric flow rate at cross-section varying along with the height with different initial parameters was depicted in Figure 8.

Figure 8 indicated that the volumetric flow rate was increasing continuously along with the height due to entrainment on ambient air. It was assumed that the local rate of entrainment consisted of two components; one was the component of entrainment due to jet momentum while the other was the component of entrainment due to buoyancy. The volumetric flow rate of high-temperature annular buoyant jets at cross-section increased with the initial temperature increasing at a constant initial velocity except for Case 3 and with the initial velocity increasing at a constant initial temperature. The special development laws of Case 3 would be explained as follows.

**Figure 9:**
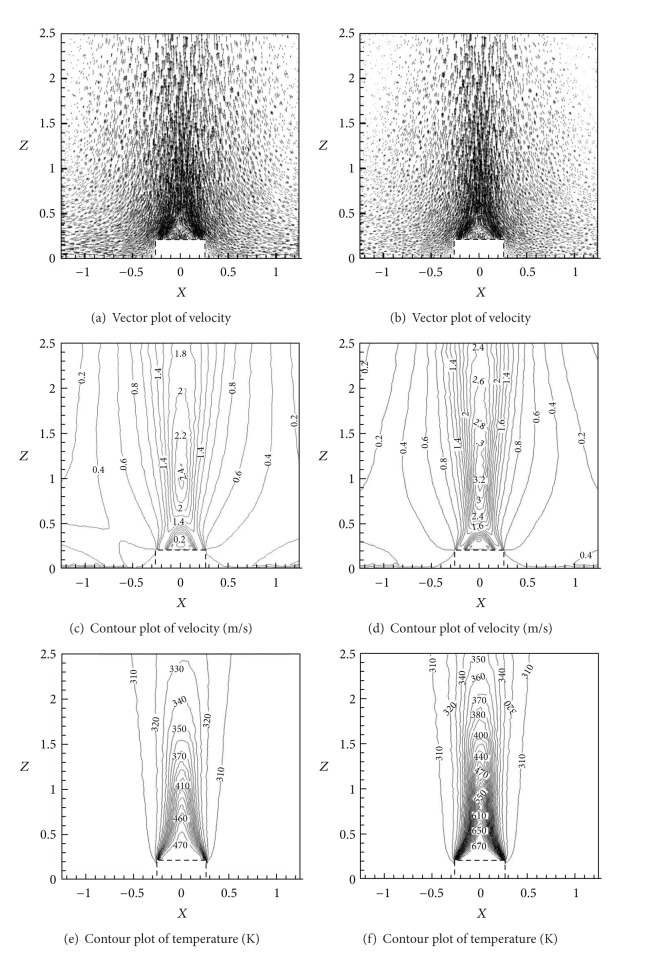
Comparison of velocity and temperature field between Case 2 (*U*_0_ = 1.2 m/s, *t*_0_ = 200°C) and Case 3 (*U*_0_ = 1.2 m/s, *t*_0_ = 400°C).

The predominant effect of positive thermal buoyancy was to increase the axial velocity component of the jet. In Case 3, the buoyancy force was much stronger than the inertia force due to sharp temperature difference between axial temperature and ambient temperature. At this moment, the development law of annular buoyant jet was similar to that of pure plume. The density of buoyant jets decreased and so did the pressure. The buoyant jet was extruded intensively by the ambient air, resulting in sharp contraction of cross-section and sharp decrease of volumetric flow rate. The comparison of flow-field characteristics between Case 2 (left) and Case 3 (right) might explain the phenomenon clearly, as shown in Figure 9.

#### 3.2.3. Development Laws of Cross-Section Diameter with Different Initial Parameters

**Figure 10:**
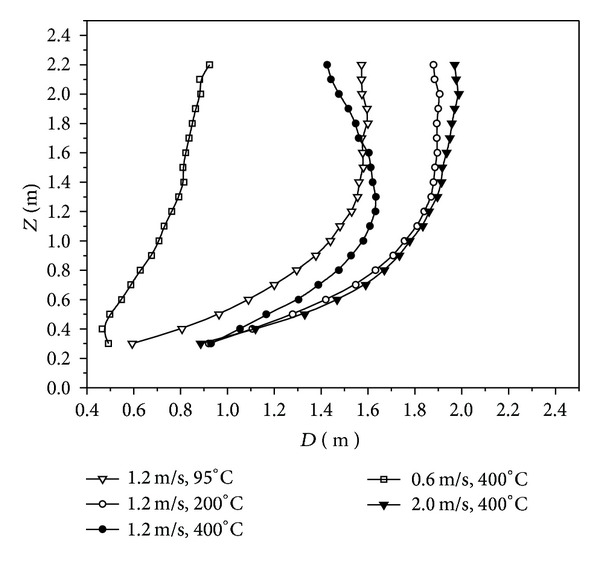
Cross-section diameter of high-temperature annular buoyant jets varying along with the height with different initial parameters.

The cross-section diameter, *D*, of high-temperature annular buoyant jets varying along with the height with different initial parameters accorded with that of volumetric flow rate, as shown in Figure 10.

The exhaust hood shall be installed at a small cross-section diameter and volumetric flow rate of high-temperature annular buoyant jet. By this way, both the size and air volume of the exhaust hood could be reduced to improve the control efficiency and reduce the energy consumption. Therefore, the exhaust hood should be selected and installed reasonably on the basis of having good knowledge of the development laws of cross-section diameter and volumetric flow rate of high-temperature buoyant jets.

### 3.3. Influence of Pressure at Exhaust Hood Inlet on Flow Field of High-Temperature Annular Buoyant Jets

**Figure 11:**
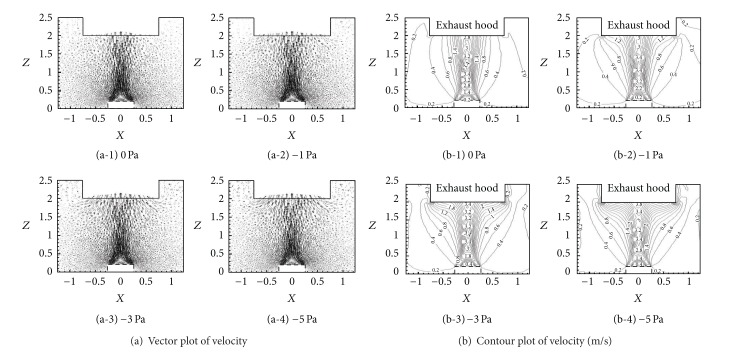
Velocity field characteristics of high-temperature annular buoyant jets with different pressures at the exhaust hood inlet.

Install the square exhaust hood on the aforementioned basic model. The size of the exhaust hood was 1.5 m × 1.5 m × 0.5 m, and it was installed at 2 m height (seen in Figure 1). The initial velocity of high-temperature annular buoyant jets was 1.2 m/s, and the initial temperature was 400°C. The pressure at the exhaust hood inlet had a great impact on the velocity field characteristics. They were set as 0 Pa, −1 Pa, −3 Pa, and −5 Pa to compare the differences. However, the temperature field and pressure field had no significant change with the increasing pressure at the exhaust hood inlet. The velocity field characteristics of high-temperature annular buoyant jets with different pressures at the exhaust hood inlet were shown in Figure 11.

The following conclusions could be drawn from Figure 11. The maximum axial velocity of high-temperature annular buoyant jets was increasing with the increasing pressure at the exhaust hood inlet. When the pressure at the exhaust hood inlet was 0 Pa, the maximum axial velocity occurred at the middle of the annulus and the exhaust hood inlet. And the streamlines had approximately the same direction around the exhaust hood inlet. When it was −1 Pa, the maximum axial velocity occurred at the approximately same position and the airflows were inhaled into the exhaust hood in disorder. The maximum axial velocity occurred not only at the middle of the annulus and the exhaust hood inlet but also around the exhaust hood inlet when it was changed to −3 Pa. However, the maximum axial velocity occurred around the exhaust hood inlet when it reached −5 Pa. The velocity field characteristics of annular buoyant jets were pretty similar when the negative pressure at the exhaust hood was smaller than −5 Pa. In a word, the airflow could be effectively controlled without great passive pressure at the exhaust hood inlet.

## 4. Conclusions

The aim of this study was to numerically investigate the flow characteristics of high-temperature annular buoyant jets and the air distribution characteristics under the coupling effect of high-temperature annular buoyant jets and ventilation system. Based on the analysis of the simulation results and comparisons with previous studies, the following remarkable conclusions were drawn.

(1) Two peak velocities occurred in the process of high-temperature annular buoyant jets development with the ratio of outer diameter to inner diameter of the annulus smaller than 5/2. The middle peak velocity was mainly caused by the vortex with buoyant jets at the sides expanding to the center. In contrast, peak velocities at the sides occurred when acceleration action due to buoyancy and velocity decay due to entrainment were in equilibrium. The middle peak velocity gradually decreased and disappeared eventually with the increase of width of high-temperature annular buoyant jets.

(2) Radial velocity developments of high-temperature annular buoyant jets with different annular jet widths revealed that the annular flow merged towards the axis of the annulus forming a velocity profile further downstream similar to that for a circular jet.

(3) When the ratio of outer diameter to inner diameter of the annulus was smaller than 5/2, the flow-field characteristics of high-temperature annular buoyant jets had significant difference compared to circular buoyant jets with the same outer diameter. In this case, the high-temperature annular buoyant jets could not be simplified as circular buoyant jets.

(4) The reattachment points for ratio of outer diameter to inner diameter of 5/4, 5/3, 5/2, and 5/1 correspondingly occurred at *Z*/*D*_0_ = 2.60, 2.40, 1.90, and 1.80. For similar diameter ratios, reattachments in this paper occurred further downstream in contrast to previous study. This phenomenon might be due to the strong buoyancy force effects on the hot air jets.

(5) The volumetric flow rate was increasing continuously along with the height due to entrainment on ambient air. The cross-section diameter of high-temperature annular buoyant jets varying along with the height with different initial parameters accorded with that of volumetric flow rate.

(6) When the pressure at the exhaust hood inlet changed from 0 Pa to −5 Pa, the position where maximum axial velocity occurred was moving from the middle of annulus and exhaust hood inlet towards the exhaust hood inlet. The velocity field characteristics were pretty similar when the negative pressure at the exhaust hood was greater than −5 Pa.

## Figures and Tables

**Table 1:** Initial parameters of annular buoyant jets.

Cases	Initial velocity	Initial temperature	*D*_*i*_	*D*_0_
	(m/s)	(°C)	(m)	(m)
1	1.2	95	0.3	0.5
2	1.2	200	0.3	0.5
3	1.2	400	0.3	0.5
4	0.6	400	0.3	0.5
5	2.0	400	0.3	0.5
